# Analysis of Cardiac Vibration Signals Acquired From a Novel Implant Placed on the Gastric Fundus

**DOI:** 10.3389/fphys.2021.748367

**Published:** 2021-11-19

**Authors:** Henry Areiza-Laverde, Cindy Dopierala, Lotfi Senhadji, Francois Boucher, Pierre Y. Gumery, Alfredo Hernández

**Affiliations:** ^1^Univ Rennes, INSERM, LTSI - UMR 1099, Rennes, France; ^2^SentinHealth SA, Biopolis, Grenoble, France; ^3^Univ. Grenoble Alpes, CNRS, Grenoble INP, TIMC-IMAG, Grenoble, France

**Keywords:** cardiac vibration signals, seismocardiogram (SCG), implantable devices, biomedical signal processing, heart failure

## Abstract

The analysis of cardiac vibration signals has been shown as an interesting tool for the follow-up of chronic pathologies involving the cardiovascular system, such as heart failure (HF). However, methods to obtain high-quality, real-world and longitudinal data, that do not require the involvement of the patient to correctly and regularly acquire these signals, remain to be developed. Implantable systems may be a solution to this observability challenge. In this paper, we evaluate the feasibility of acquiring useful electrocardiographic (ECG) and accelerometry (ACC) data from an innovative implant located in the gastric fundus. In a first phase, we compare data acquired from the gastric fundus with gold standard data acquired from surface sensors on 2 pigs. A second phase investigates the feasibility of deriving useful hemodynamic markers from these gastric signals using data from 4 healthy pigs and 3 pigs with induced HF with longitudinal recordings. The following data processing chain was applied to the recordings: (1) ECG and ACC data denoising, (2) noise-robust real-time QRS detection from ECG signals and cardiac cycle segmentation, (3) Correlation analysis of the cardiac cycles and computation of coherent mean from aligned ECG and ACC, (4) cardiac vibration components segmentation (S1 and S2) from the coherent mean ACC data, and (5) estimation of signal context and a signal-to-noise ratio (SNR) on both signals. Results show a high correlation between the markers acquired from the gastric and thoracic sites, as well as pre-clinical evidence on the feasibility of chronic cardiovascular monitoring from an implantable cardiac device located at the gastric fundus, the main challenge remains on the optimization of the signal-to-noise ratio, in particular for the handling of some sources of noise that are specific to the gastric acquisition site.

## 1. Introduction

Patients suffering from chronic pathologies involving the cardiovascular system, such as heart failure (HF), may benefit from a long-term remote monitoring of the main cardiovascular parameters in order to early diagnose decompensation events or to adapt their therapy in a personalized and continuous fashion (Cleland et al., 2006; Desai et al., 2017). The analysis of cardiac vibration signals, interesting sources of information about the cardiac mechanical activity, has already shown remarkable results in this context, mainly because the main components of these signals have been associated with some useful hemodynamic markers (Plicchi et al., 2002; Bordachar et al., 2008). Further, Micro-Electro-Mechanical Systems (MEMS) sensor technology, commonly used to acquire cardiac vibration signals, have improved significantly during the last two decades in terms of size, cost, and resolution.

Cardiac vibration signals may be acquired non-invasively from the chest, in a similar fashion as cardiologists apply the stethoscope for listening to the phonocardiogram (PCG) (Jain and Tiwari, 2014). The acquisition of accelerometry signals from the chest of the patient, using in particular these MEMS devices, leads to the observation of the seismocardiography (SCG) signal, that is characterized by the presence of two main components, S1 and S2, which correspond to the first and second heart sounds in the PCG, respectively. SCG acquisition and processing have been widely developed during the last decade, with applications ranging from coronary artery disease characterization, to cardiac stress and heart failure monitoring (Inan et al., 2014). In Donal et al. (2011) and Giorgis et al. (2012), a number of features were extracted from chest accelerometry signals and compared to classical hemodynamic echocardiography markers, in order to optimize parameters of cardiac resynchronization therapy devices implanted on HF patients. Furthermore, recent developments of wearable or connected devices offer the possibility to monitor the cardiac vibration signals in ambulatory monitoring (Shandhi et al., 2019; Gupta et al., 2020). However, methods to obtain high-quality, chronic and longitudinal cardiac vibration data, that do not require the involvement of a medical practitioner or the patient to correctly and regularly acquire these signals, remain to be developed.

Implantable systems may be a solution to this observability challenge. A number of studies have been focused on the acquisition of accelerometric signals to measure cardiac vibrations signals from inside the heart chambers in an invasive manner (Hernández et al., 2013; Gallet et al., 2016). These endocardial acceleration (EA) signals have two main components known as EA1 and EA2, which are associated with the first and second heart sounds, respectively. The peak-to-peak values of the main cardiac vibration components of these signals have been shown to be significantly correlated with meaningful hemodynamic markers. Results shown by Plicchi et al. (2002) demonstrated a significant correlation between the peak-to-peak value of EA1 and the positive peak of the first derivative of the left ventricular (LV) pressure, *dP*/*dt* (*r* = 0.83, *P* < 0.001), between the peak-to-peak value of EA2 and the negative peak of LV *dP*/*dt* (*r* = 0.92, *P* < 0.001) and the peak-to-peak value of EA2 with aortic diastolic pressure (*r* = 0.91, *P* < 0.001). All these experiments were developed at baseline and during different acute hemodynamic interventions. Such results were further confirmed by Bordachar et al. (2011), obtaining a significant correlation between the peak-to-peak value of EA1 and the positive peak of LV *dP*/*dt* (*r* = 0.91, *P* < 0.001). Additionally, Bordachar et al. (2008) evaluated the correlation between the peak-to-peak value of S1 and LV *dP*/*dt* (*r* = 0.93, *P* < 0.001), reaffirming the close relationship between EA and surface cardiac vibration signals. These relationships demonstrate the usefulness of cardiac vibration signals to infer and monitor hemodynamic parameters in a more practical and simpler but precise way.

Implantable cardiac devices (ICD) such as cardiac resynchronization therapy defibrillators, cardioverter-defibrillators and pacemakers are normally used in the treatment and follow-up of chronic heart diseases as heart failure (HF). Some of these ICD already integrate accelerometer sensors to observe and analyze cardiac accelerometry signals (ACC) from subcutaneous or intra-cardiac sites, with the objective to predict future HF events (Boehmer et al., 2017; Cao et al., 2020) or to define automatic calibration protocols of the ICD parameters (Delnoy et al., 2008; Hernández et al., 2013). Additionally, the increasing study of cardiac vibration signals for the development of ICD in the field of HF has led to the proposal of candidate markers of the progression this disease, such as the presence of an S3 component (Siejko et al., 2013; Thakur et al., 2017), and the expansion of the analysis of these signals toward the multimodal field by using 3D accelerometers (Calvo et al., 2018).

In past works, our group has proposed methods for the acquisition and processing of cardiac mechanical signals from accelerometers embedded into the stimulation lead of cardiac implantable devices (Hernández et al., 2013; Gallet et al., 2016; Calvo et al., 2018). Although these developments have provided interesting results for the optimal handling of cardiac resynchronization therapy in HF patients, not all patients may benefit from an active cardiac implantable device (Ponikowski et al., 2016; Yancy et al., 2017). Hence, the development of a remote cardiac vibrations monitoring system offering integrated management of multimodal parameters with a minimally invasive device is currently needed in the chronic cardiovascular diseases domain, to trigger very early and adequate medical attention against the decompensation events.

This work explores the hypothesis that a cardiac vibration signal may be captured from a small monitoring implant, positioned at the gastric fundus, which could be delivered through gastroscopy, in a minimally invasive manner. This anatomical site seems a good candidate location for a long-term cardiovascular monitoring since it is physically close to the heart. However, ECG and ACC data acquired from this site may have specific characteristics and noise conditions, particularly related to the location and orientation of the device, as well as the interference from the electrical and mechanical activities of the gastric system. The objective of this paper is thus to characterize ECG and ACC data acquired from the gastric fundus in pre-clinical experimentation and to evaluate the feasibility to obtain useful hemodynamic markers from these signals.

## 2. Methods

This work is divided into two phases: Phase 1 is focused on the comparison of the signals acquired from the gastric fundus with data acquired through standard sensors from thoracic sites. A set of cardiovascular markers are extracted from both the reference and gastric signals and quantitatively compared. Phase 2 was dedicated to the evaluation of the feasibility of the estimation of longitudinal cardiovascular markers from the gastric site.

### 2.1. Data Acquisition

#### 2.1.1. Description of the Implantable Device

**Figure 1:**
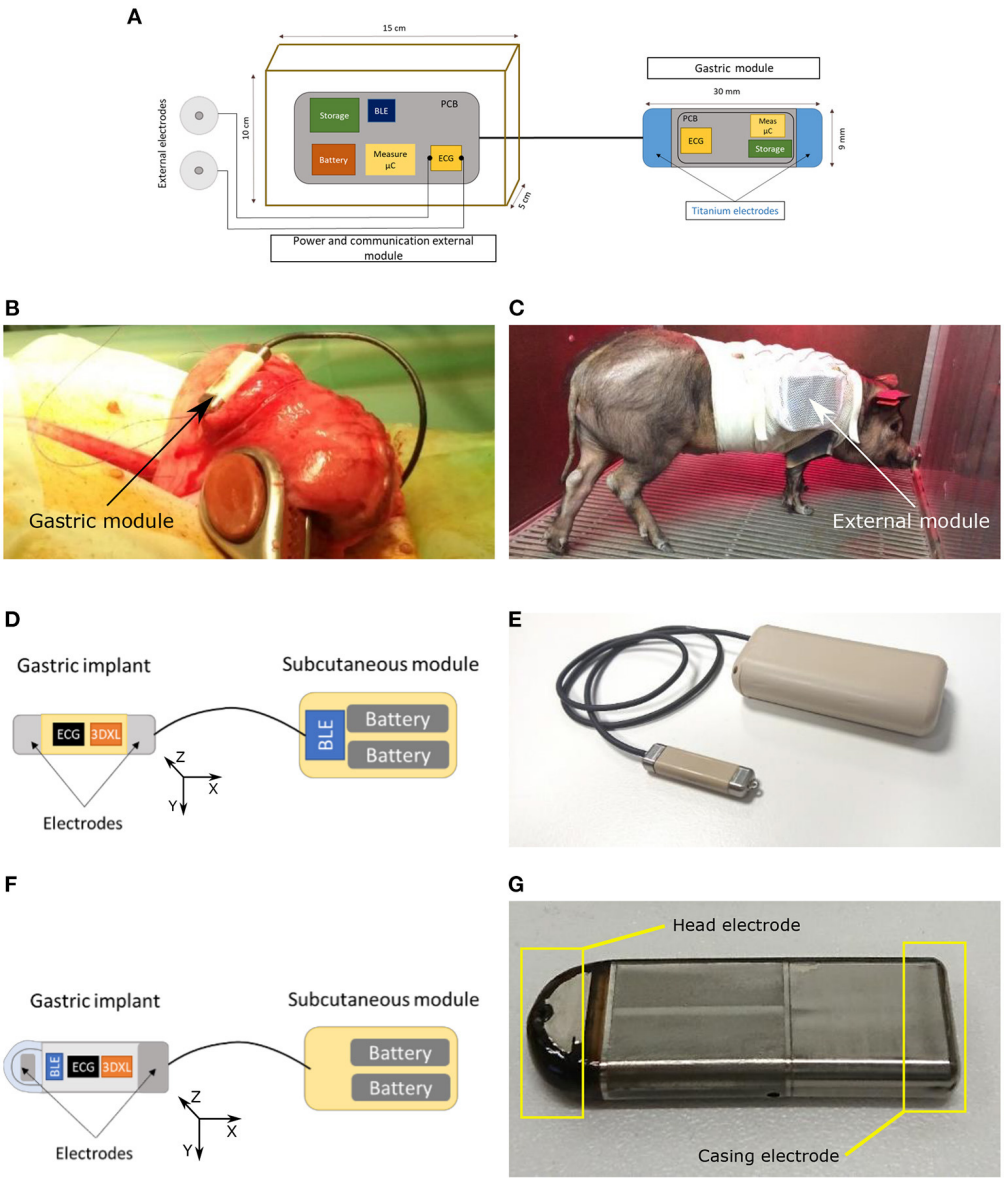
Implant prototypes used for acquiring the ECG and ACC data. **(A)** Schematic representation of the implant prototype V0. **(B)** Physical design of the gastric implant prototype V0. **(C)** Physical design of the external module of prototype V0. **(D)** Schematic representation of the implant prototype V1. **(E)** Physical design of the implant prototype V1. **(F)** Schematic representation of the implant prototype V2. **(G)** Physical design of the gastric module of the implant prototype V2.

Most gastric implants currently available are prescribed to treat gastric dysmotility syndromes and obesity. A number of works have shown the safety and tolerance of these devices, as well as recent developments on minimally invasive techniques to deliver these implants, improving patient comfort and adherence (Hasler, 2009; Carrano et al., 2020). The SentinHealth company recently developed an innovative gastric implant prototype for acquiring electrophysiological and mechanical cardiac data from the gastric fundus (Dopierala et al., 2019). This implant may be as well-tolerated as other gastric implants and could be delivered using similar minimally invasive implanting techniques. Three prototype versions of the device have been used in this work: one semi-implantable version (V0) and two fully implantable versions (V1 and V2):

- Prototype V0 (Figure 1A) is the only semi-implantable version of the device. It is made up of two modules: (1) a gastric module 30 mm long, 9 mm wide, 7 mm high, with each end corresponding to a titanium electrode having 35 mm^2^ surfaces and 20 mm distance between electrodes. Electrodes are connected to an ECG chip embedded into the capsule (Figure 1B). (2) A reference ECG module composed of two external electrodes attached to the thorax: one on the anterior thorax and the other on the posterior thorax. These electrodes are connected to an external electronic board encased in a pig jacket (Figure 1C). Both ECG signals are acquired synchronously with a 1 kHz sampling frequency.
- Prototype V1 (Figures 1D,E) consists of two interconnected, implantable modules: the first module (located in the gastric fundus) is 35 × 9 × 5.5 mm and embeds a 3D accelerometer as well as an ECG acquisition device with two titanium electrodes, with the same geometry as prototype V0, located at each end of the capsule and electrically isolated from each other by a body in PEEK. The second module, subcutaneously implanted in the abdomen, is 70 × 30.5 × 16 mm and is connected to the first module by a wire of 30 cm. It embeds two AA batteries as well as the Bluetooth low energy chip (2.4 GHz) that allows to communicate data to an external gateway for further processing. ECG sampling rate in this version is 1 kHz while ACC sampling rate is 4 kHz acquired in the bandwidth 0–1 kHz. The gastric module weight is 3.3 g.
- Prototype V2 (Figures 1F,G) differs from the previous one by two main aspects: firstly, the gastric capsule geometry, size and material were redesigned to be closer to a final device built in one piece (for future development). One of the electrodes is a part of the casing that is made in titanium. To ensure an electrical isolation of the rest of the casing, a coating with epoxy resin was applied at the surface of the titanium. The other electrode is an isolated titanium part (see Figure 1E). Secondly, the communication components have been transferred inside the gastric module. For that, a specific antenna was designed to optimize the performance of Bluetooth transmission from the stomach. The antenna is located at one end of the gastric capsule and overmolded in epoxy resin. The geometry and material of the subcutaneous module has not been changed from the V1. This module embeds only the AA battery. Regarding technical aspects, the gastric module in this version is 40 × 13.5 × 5.5 mm and its weight is 7 g. Also the ECG chip was changed to optimize the current consumption with a sampling rate of 498 Hz.

The implant rests in a low battery consumption mode while not measuring any signal, in order to extend the battery life and is programmed to automatically switch to the active mode for a duration of 30 s, in order to acquire the simultaneous ECG and ACC signals. The 30 s duration has been selected as a good compromise between energy consumption and the minimal number of cardiac cycles required to obtain a stable representation of the mean cardiac cycle observed from the intragastric ECG and ACC signals. Furthermore, the final device is intended to embed battery in a first generation. Next generation of the device aims to embed a rechargeable battery to be powered wirelessly, in order to increase the lifespan and the patients follow-up.

#### 2.1.2. Data Acquired for Phase 1

In order to validate the measurements taken from the gastric site, ECG and ACC data were simultaneously acquired from standard thoracic locations, used as gold-standard, and the proposed gastric location using two experimental setups:

1. Setup 1 was focused on the validation of ECG data. 459 recordings each one of 30 s duration were acquired using the V0 prototype over a 14-day period, from one pig. Each recording consists of synchronous data from a standard bipolar surface ECG (gold standard reference) and a gastric ECG.
2. Setup 2 was focused on the validation of ACC data. Two recordings were acquired simultaneously from a second pig, with prototype V1 and an external digital stethoscope (3M™ Littmann, USA), used as a gold standard PCG. A Valsalva-like respiratory maneuver was applied during data acquisition in order to evoke hemodynamic modifications that can be observable from both acquisition sites. Data acquisition was performed acutely, under anesthesia, with ventilator-assisted respiration and consisted of one stage of continuous positive inspiratory pressure (CPP) of 30 cmH_2_O of 15 s duration, followed by a 10 s apnea at atmospheric pressure (Gallet et al., 2016). The main objective of this setup was to validate if the evolution of the markers obtained from the gastric and reference sites during the Valsalva-like maneuver are correlated.

#### 2.1.3. Data Acquired for Phase 2

Implant prototypes V1 and V2 were used to acquire data from the gastric fundus of 4 healthy pigs and 3 pigs with induced chronic ischemic HF, leading to acute decompensated HF. Each system has been implanted for a minimal duration of one week, with a maximum of 2 weeks. During this period, animals are kept within individual cages with controlled temperature and normal feeding conditions. Caregivers take care of pigs daily to ensure that they are healthy and the device does not induce any problem such as pain or loss of appetite. The gateway is placed above cages at a distance of about 1 m to the pig. Data are recorded from the implant during 30 s every hour, with random acquisition cessation periods related to some technical problems (sometimes the scheduled acquisition was not performed or sometimes the device was not able to communicate with the server). The whole acquired database results in a total of 999 30-s recordings, with the distribution shown in **Table 3**.

All animal experiments were previously submitted to an ethics committee, in accordance with the French regulation and conducted in specialized structures with dedicated site approval, by a team composed of qualified staff who has completed regulatory training in animal testing and experimental surgery.

### 2.2. Data Processing

**Figure 2:**
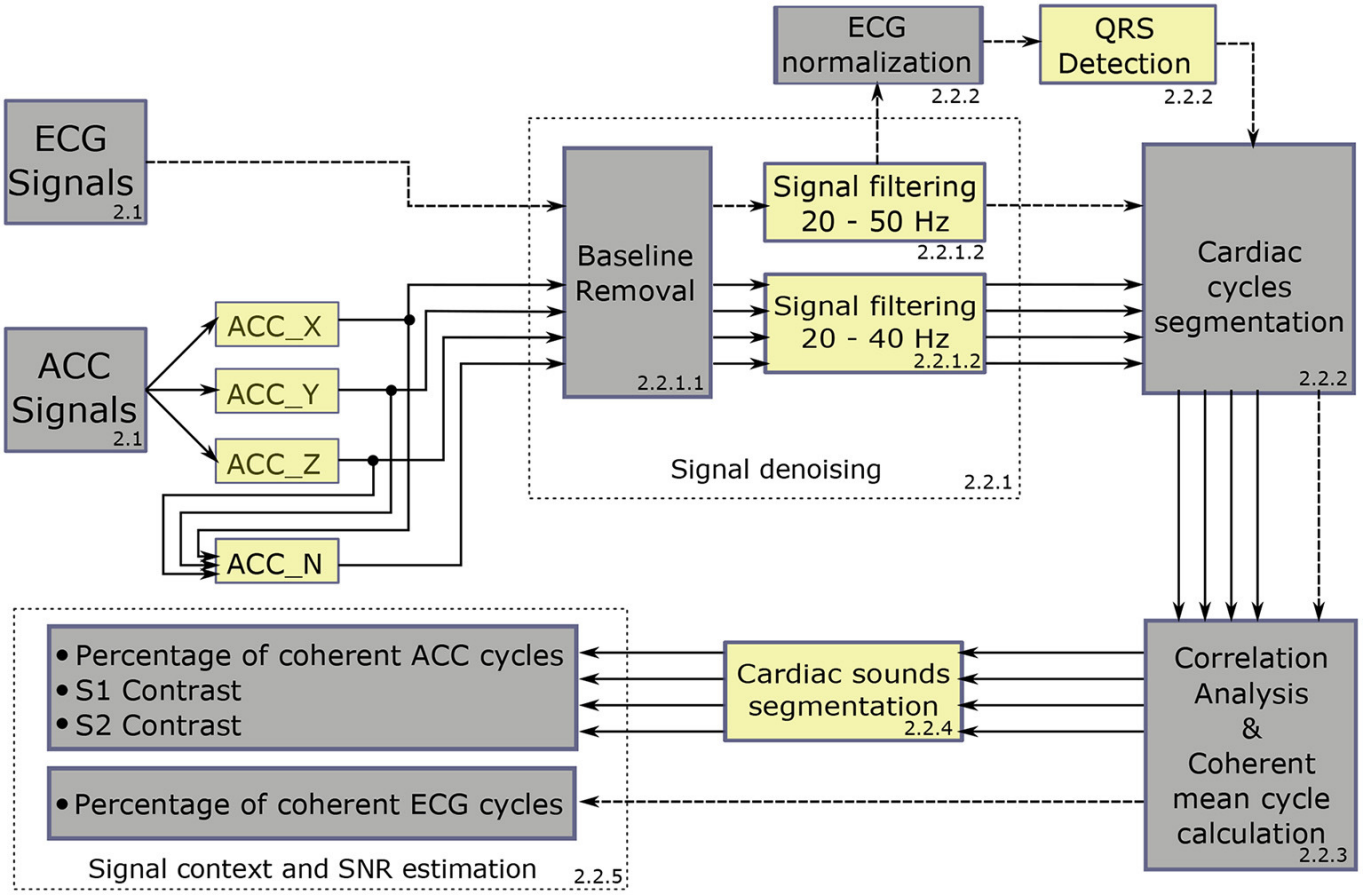
Global diagram of the processing chain applied to the acquired data. Dashed-line arrows represent the ECG signal pipeline and solid-line arrows represent the ACC axes pipeline.

Figure 2 summarizes the signal processing chain applied to the cardiac data: (1) ECG and ACC data denoising, (2) noise-robust real-time QRS detection from ECG signals and cardiac cycle segmentation, (3) correlation analysis of the cardiac cycles and computation of the coherent mean from aligned ECG and ACC segmented cycles, (4) segmentation of cardiac vibration components (S1 and S2) from the coherent mean ACC data, and (5) estimation of signal context and a signal-to-noise ratio (SNR) on both signals.

#### 2.2.1. ECG and ACC Data Denoising

##### 2.2.1.1. Baseline Removal

ECG and ACC signals are affected by low-frequency noise from two main different sources. The first noise source is from the instrumentation. Indeed, each transition from the low consumption mode to the active mode generates a low-frequency transitory state during the first 5 s of data acquisition. In order to exploit as much of the signal as possible, a baseline removal process is applied to the signals, managing to process 29 s from each signal, and having to remove just the first second, where the transitory state may saturate the amplifiers and completely impede the acquisition of any useful data. The second source of noise are the electrophysiological and mechanical components of electrogastrographic and respiratory activity, that are captured by the electrodes and the accelerometer.

The baseline removal process is thus applied to ECG and ACC data and is based on a locally weighted linear regression algorithm (Šarlija et al., 2017). In order to reduce the computational cost of the baseline removal process, the linear regression algorithm is applied over 4-s windows and each signal is downsampled to 400 Hz in each window. The linear regression algorithm output is the baseline representation of the signal, which is directly subtracted from the original signal, preserving its main features (Šarlija et al., 2017). The norm of the 3D ACC vector is computed before applying the baseline removal process, and it is treated as a new ACC axis from this point, by representing an ACC component independent of the direction of acceleration.

##### 2.2.1.2. Signal Filtering

**Figure 3:**
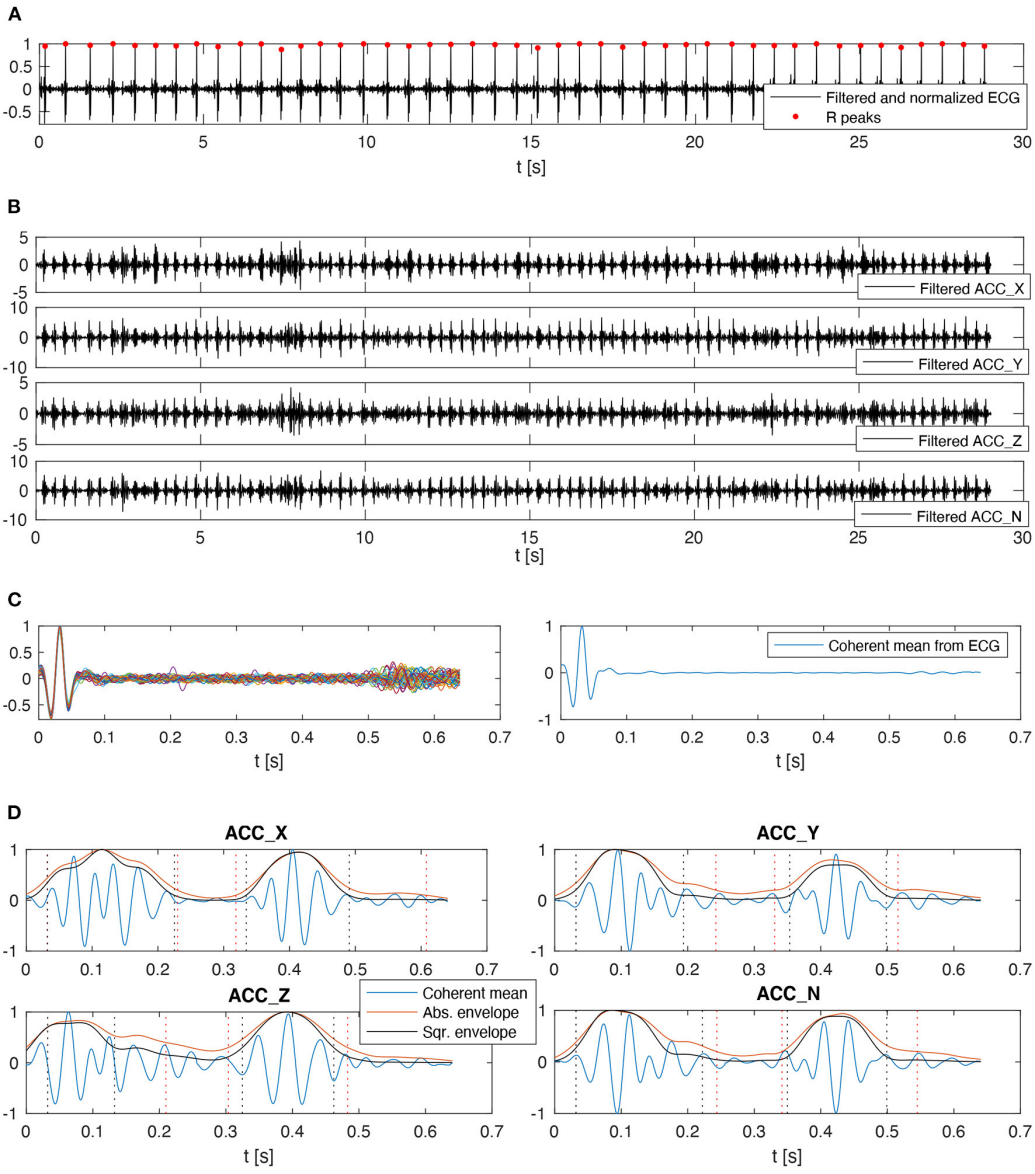
Data processing chain applied to the electrophysiological and mechanical cardiac data. **(A)** ECG signal after applying baseline removal, filtering, normalization, and QRS detection processes. **(B)** ACC axes after applying the baseline removal and filtering processes, the N-axis represents the norm. **(C)** Segmented and aligned ECG cardiac cycles in the left, and the corresponding coherent mean cardiac cycle in the right. **(D)** Coherent mean cardiac cycle on each ACC axis with their respective envelopes, including candidate detections for S1 and S2. The vertical dotted lines represent *t*1, *t*2, *t*3, and *t*4 in red color for Abs and black for Sqr.

Filtering methods are widely documented in the literature for ECG and cardiac vibration signal analysis. The combination of independent high-pass and low-pass fifth-order Butterworth filters is applied to the signals using zero-phase forward and reverse digital IIR filtering, and defining different cutoff frequency values concerning the signal type. The selected band for the ECG signals is 20–50 Hz in order to reduce the amplitude of the T-wave and emphasize the R peak to facilitate the subsequent QRS detection process. The band for the ACC signals is 20–40 Hz, considering the frequency bands that contain most of the energy of the signal in local cardiac accelerometer signals (Giorgis et al., 2012; Cordero Álvarez, 2020). Figures 3A,B show representative examples of filtered ECG and ACC signals, respectively.

#### 2.2.2. QRS Detection From ECG Signals

Aiming to facilitate the QRS detection process, a locally conducted normalization based on the local minima/maxima values was applied to the ECG filtered signal. This normalization algorithm is presented and described in detail by Šarlija et al. (2017). The only modification applied in this work to the original normalization algorithm is the use of the median value instead of the mean for clipping the lowest values of the normalization signal. This change corresponds to the robustness presented by the median value to outliers, improving the algorithm stability even against some particularly noisy signals. After normalizing the ECG signal, a robust, real-time QRS detector based on a multi-feature probabilistic method was used to identify the R peak positions from the ECG signal (Doyen et al., 2019). This detector was used because the target signals were suspected to be highly-artifacted, and it was necessary to handle them with a detector designed for such conditions. Figure 3A shows one example of the normalized ECG with its respective detected R peaks.

After applying the QRS detection algorithm to the ECG signals, the heart rate is calculated by using the median duration of all cardiac cycles. The cardiac cycles are segmented by taking 0.05*60/*HR* seconds before the R peak as the starting point of each cardiac cycle. It is a dynamic delay established to preserve the complete waveform of the QRS complex, independently of the heart rate variation among the signals in the whole dataset. The cardiac cycle segmentation process is directly applied to the ECG signal and subsequently projected to all axes of the ACC signal because both signals were simultaneously acquired.

#### 2.2.3. Correlation Analysis of Cardiac Cycles

Once the cardiac cycle start points are defined, the median cycle duration is used to resize all the cycles through a zero-padding technique. This applies to both ECG and ACC signals. Later, in the case of ECG signals, the normalized cross-correlation between each pair of cycles is computed, and the dominant group of cycles with a correlation coefficient higher than 0.6 is used to compute a coherent mean cardiac cycle. It is possible to directly apply this process because all the ECG cycles are aligned by the occurrence time of each R peak and the result of the maximum correlation value. Figure 3C shows an example of the ECG cardiac cycles aligned and the corresponding coherent mean cardiac cycle.

The correlation analysis for the ACC signals is slightly more complex because the exact starting point of S1 and S2 vary independently of the occurrence time of the R peak, mainly due to beat-to-beat modifications of the inotropic state and the preload and afterload conditions. Therefore, it is necessary to apply independent phase correction stages for S1 and S2 in order to increase the inter-cycle correlation at the moment of calculating the coherent mean cardiac cycle (Donal et al., 2011; Giorgis et al., 2012). After applying the phase optimization to each ACC cycle, the dominant group of cycles with a correlation coefficient higher than 0.6 is used to compute the coherent mean cardiac cycle, independently over each ACC axis. Considering the ACC signals can not be normalized because it would affect the relation between the cardiac vibration components and the hemodynamic markers, outlier cycles are removed according to their energy (an outlier is a value that is more than three scaled median absolute deviations away from the median), aiming to remove the cycles which contain particular noises, such as pig growls, gastric sounds, or vibrations that could disturb the coherent mean cycle calculation.

#### 2.2.4. Cardiac Sound Segmentation (S1 and S2)

Giorgis et al. (2012) proposed an effective algorithm to estimate the timings of S1 and S2 using the coherent mean of the ACC signals. Firstly, the coherent mean cycle is normalized, and the absolute (Abs) and squared (Sqr) envelopes are computed. Then, a dynamic threshold between 0.1 and 0.7 is used to identify S1 in the first half of the cycle. S1 is detected by searching the points where Abs or Sqr cross the threshold (fixing the R-peak time as the earliest possible time to define the start point of S1). Similarly, S2 is identified in the cycle segment between the end of S1 and the end of the cycle. The result of implementing this algorithm is the starting and ending times of S1 and S2. These values can be defined as follows:

- *t*0 = Reference instant for the start of cardiac cycle (obtained from ECG).
- *t*1 = Start of S1
- *t*2 = End of S1
- *t*3 = Start of S2
- *t*4 = End of S2
- *t*5 = End of cardiac cycle

Hence, S1 corresponds to the signal segment between *t*1 and *t*2, S2 corresponds to the signal segment between *t*3 and *t*4, and finally, the union of the signal segments *t*2-*t*3 and *t*4-*t*5 are considered as the signal background. This process is applied over each ACC axis, as well as the norm. An example of the result of this process is shown in Figure 3D.

#### 2.2.5. Signal Context Estimation and SNR

Aiming to quantitatively assess the quality of the signals, different features are considered to estimate the SNR. Based on the context variables presented by Giorgis et al. (2012) to setting the control algorithm that automatically recognizes the context of the ACC signals to segment S1 and S2, the following quality measures are proposed in this work:

- The percentage of coherent ECG cycles.
- The percentage of coherent ACC cycles over each axis.
- The S1 contrast, defined as the ratio between the standard deviation of S1 and the standard deviation of the signal background.
- The S2 contrast, defined as the ratio between the standard deviation of S2 and the standard deviation of the signal background.

In order to estimate the general data quality of each recording, three successive classification stages were applied, using the quality measures defined above.

**Figure 4:**
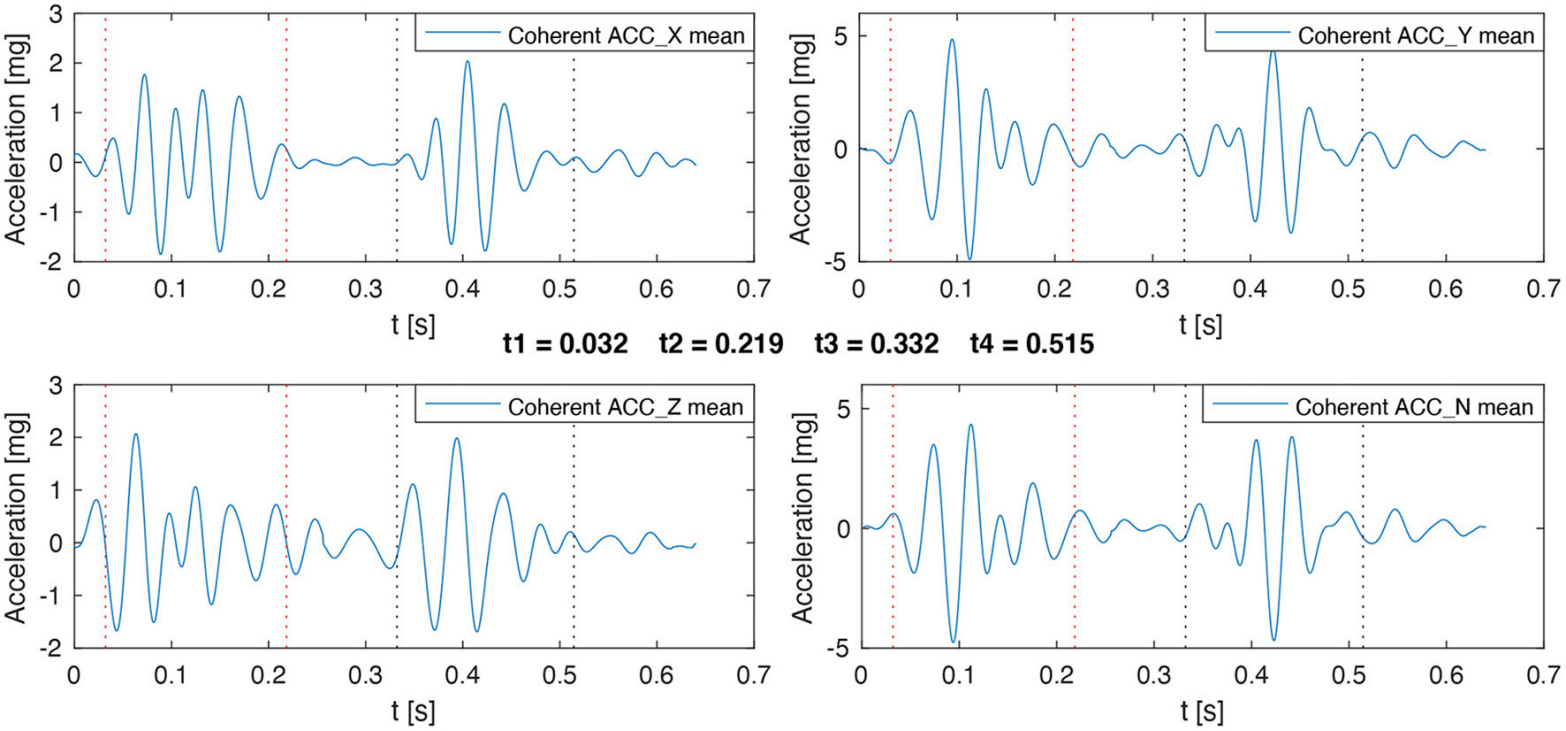
Example of the final detection instants estimated for S1 and S2 on a representative recording. Vertical dotted lines represent the start and the end of S1 in red and S2 in black.

1. The first stage is related to the analysis of the coherent cardiac cycles in the ECG and ACC signals. Indeed, in stable cardiovascular conditions and sinus rhythm, the relative number of coherent cardiac cycles may be considered as a marker of signal quality. In this stage, only the recordings with three or more coherent cycles in the ECG and at least one of the ACC axis were preserved. This relatively low threshold for the number of coherent cardiac cycles was considered appropriate since two other quality assessment phases follow the classification phase.
2. The second stage is based on the detection of S1 and S2 in the ACC signals. In this stage, only the signals with S1 and S2 contrast higher than 2 were preserved. This value was selected because it means that the content of S1 and S2 can stand out over the signal background by having twice the standard deviation.
3. The third stage concerns the estimation of the duration and the peak-to-peak values of S1 and S2. Since the different envelopes will provide different S1 and S2 detection instants for each ACC axis (as shown in Figure 3D), an algorithm should be applied to fuse the local detections of each axis and envelope to obtain the final S1 and S2 detection instants. The contrast measures described above were used to define the final detection instants of S1 and S2 as follows: (1) each detection instant is replicated on the four ACC axes to analyze its global performance by computing the corresponding contrast of such detection over each axis. (2) The detections with a contrast value lower than 2 are discarded on the corresponding axis. (3) Two relevance vectors are created with the contrast measures, one for S1 and another for S2, where the higher the contrast measure, the higher the relevance value assigned to the corresponding detection. (4) A weighted average between the detection instants is computed by using the relevance vectors as weights. The result of this weighted average operation is used as the final detection of S1 and S2. Figure 4 shows a representative example of the final detection instants estimated for the recording shown in Figure 3D. After estimating the final values of *t*1, *t*2, *t*3, and *t*4 for each recording, the recordings for which the duration or peak-to-peak value of S1 or S2 represent an outlier were removed.

**Figure 5:**
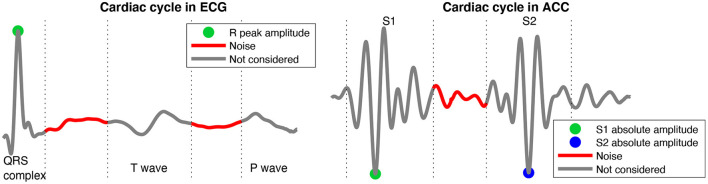
Representation of the signal of interest and noise segments of the cardiac cycle in the ECG and ACC signals to compute the SNR.

Additional SNR estimators were used to validate the quality analysis and to obtain quantitative results that can be compared with the literature. The SNR for a given heartbeat in the ECG signals is calculated by considering the power of R peak amplitude as the signal portion and the power of the segments between the QRS complex, T-wave, and P-wave as the noise-only portion of the signal, as shown in Figure 5. This analysis is performed in a similar manner on the ACC signals, by considering two different signal portions (the absolute amplitude of S1 and the absolute amplitude of S2), and the noise-only portion being the segment between S1 and S2, as shown in Figure 5. The SNR for a given heartbeat was computed using Equation (1). Then the analysis is carried out over all available heartbeats by considering an average SNR.


(1) $$\begin{array}{c} \text{SNR}=10\times \log_{10}(\frac{S^{2}}{\frac{1}{M}\sum_{m=1}^{M}|x_{m}{|}^{2}}) \end{array}$$

where *S* is the signal portion value and *x*_*m*_ is a particular sample of the total *M* samples making up the noise-only portion of the signal.

Note that ECG signals were initially filtered to reduce the amplitude of P and T-waves. In order to facilitate the comparison with other works in the literature, an alternative Band-pass filter between 5 and 50 Hz was applied to the raw ECG signals, allowing for the visualization of the T and P waves. ECG signals filtered between 20 and 50 Hz are thus noted “QRS-ECG” while ECG signals filtered between 5 and 50 Hz were noted “TP-ECG.”

### 2.3. Validation With Gold Standard References

In addition to the data processing chain explained above, other evaluation measures were applied to the surface and gastric data, with the objective of validating the gastric signals and to obtain results comparable with the literature in the field. Regarding the ECG signals, comparisons were mainly focused on QRS detection and the estimation of heart rate. In a first step, QRS detection was applied to the reference, surface signals and the obtained detections were subsequently reviewed manually to correct possible detection errors, in order to constitute the set of reference QRS instants. Quantitative QRS detection performance from the gastric site was estimated by calculating the sensitivity and the positive predictive value (+P), with respect to the QRS reference instants. A QRS detection from the implant signal is considered as true positive (TP) if it lies within a centered window of 50 ms from the corresponding reference QRS instant. All remaining QRS detections from the implant are considered as false positives (FP). False negatives (FN) occur when no detection from the gastric site is found within the matching reference window (Doyen et al., 2019). Also, the time difference between a TP detection and its corresponding reference QRS instant (Jitter) is reported. All records acquired during session 1 were used for this comparison. A Wilcoxon rank-sum test was applied to statistically compare markers obtained from the reference and gastric sites. In these analyses the level of significance was set to 0.05.

Regarding ACC signals, all measures were computed concerning S1 and S2 separately. The main objective is to compare the evolution of the derived markers acquired from the reference PCG signal with those obtained from the intragastric ACC signals during the application of the Valsalva-like maneuver. Therefore, the quantitative marker used for comparison is the correlation of the time-profiles of the main heart sound variables (duration and peak-to-peak values) with respect to those obtained from the PCG reference. The time-profiles are created by measuring the heart sound variables throughout the entire recording. The time-profiles associated with heart sound duration were computed using a sliding window of four cardiac cycles. Concerning implant ACC data, a mean cycle per axis was computed inside the window and the global duration of heart sounds was calculated after applying the algorithm described in the third stage of section 2.2.5 to estimate the final detection instants of S1 and S2, obtaining a global duration time-profile of the implant to be compared with the duration time-profile of the reference PCG. On the other hand, time-profiles of peak-to-peak values were computed independently on each ACC axis of the implant, calculating the mean of peak-to-peak values for each heart sound in a sliding window with the size of two cardiac cycles. Each peak-to-peak time-profile of the implant was compared with the time-profile obtained from the PCG reference.

## 3. Results

### 3.1. Comparison of Gastric and Thoracic Data

**Figure 6:**
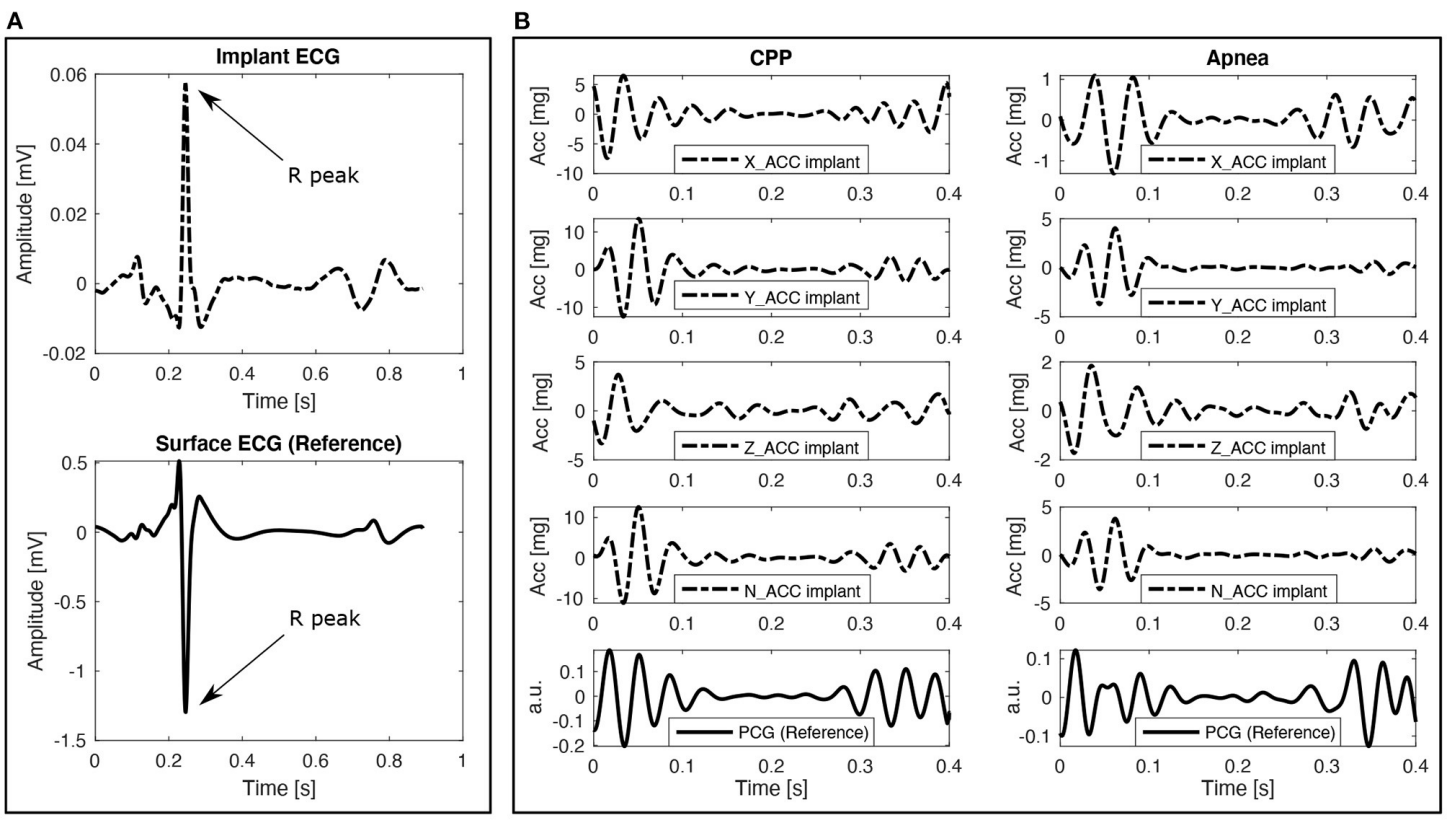
Example of implant and gold standard signals comparison. Dash-dotted lines correspond to the implant signals and solid lines correspond to the gold standard reference signals. **(A)** Coherent mean cycles of ECG signals taken from one representative recording. Note that the differences in signal morphology and amplitude between the surface and gastric devices are mainly explained by the significantly different dipoles that are observed. **(B)** Coherent mean cycles of ACC and PCG signals during both respiratory phases taken from the recording 2.

Figure 6 shows examples of data acquired from the thoracic (reference) and gastric sites for ECG (Figure 6A) and ACC (Figure 6B). ECG signals in this figure were band-pass filtered between 5 and 50 Hz and the coherent mean cycle of a representative recording is shown. The differences in signal morphology and amplitude between the surface and gastric devices are mainly explained by the significantly different dipoles that are observed. Nevertheless, even in the shorter gastric dipole, the main ECG waves are easily identifiable and a correct QRS detection for instantaneous heart rate estimation may be expected.

Table 1 shows a quantitative comparison between the ECG signals captured with the implant and the external ECG signals taken as gold standard reference. The estimated SNR are significantly lower on the implant data with respect to the reference. This is expected, due to the different noise sources associated with the gastric site. Furthermore, also significant differences are observed between the values for coherent cycles percentage and inter-cycle correlation, although both signals provided similar high and acceptable values for these markers. Finally, the sensitivity and positive predictive value for QRS detection are satisfactory, with a jitter lower than 10 ms. These results suggest that a suitable HR estimation may be performed from the gastric implant.

**Table 1:** Validation of ECG recordings with a gold standard reference.

**ECG** **Source**	**TP-ECG** **SNR [dB]**	**Rpeak-ECG** **SNR [dB]**	**Coherent cycles** **percentage [%]**	**Inter-cycle** **correlation**	**QRS detection**		
					**Sensitivity** **[%]**	**+P** **[%]**	**Jitter** **[ms]**
Surface	28.4 ± 3.4	38.4 ± 6.1	100.0 ± 0.1	0.98 ± 0.01	97.6 ± 4.7	98.2 ± 3.5	6.0 ± 3.4
Implant	21.6 ± 7.5^*^	30.3 ± 5.8^*^	99.2 ± 2.9^*^	0.94 ± 0.05^*^			

* *p < 0.05 vs. reference*.

**Figure 7:**
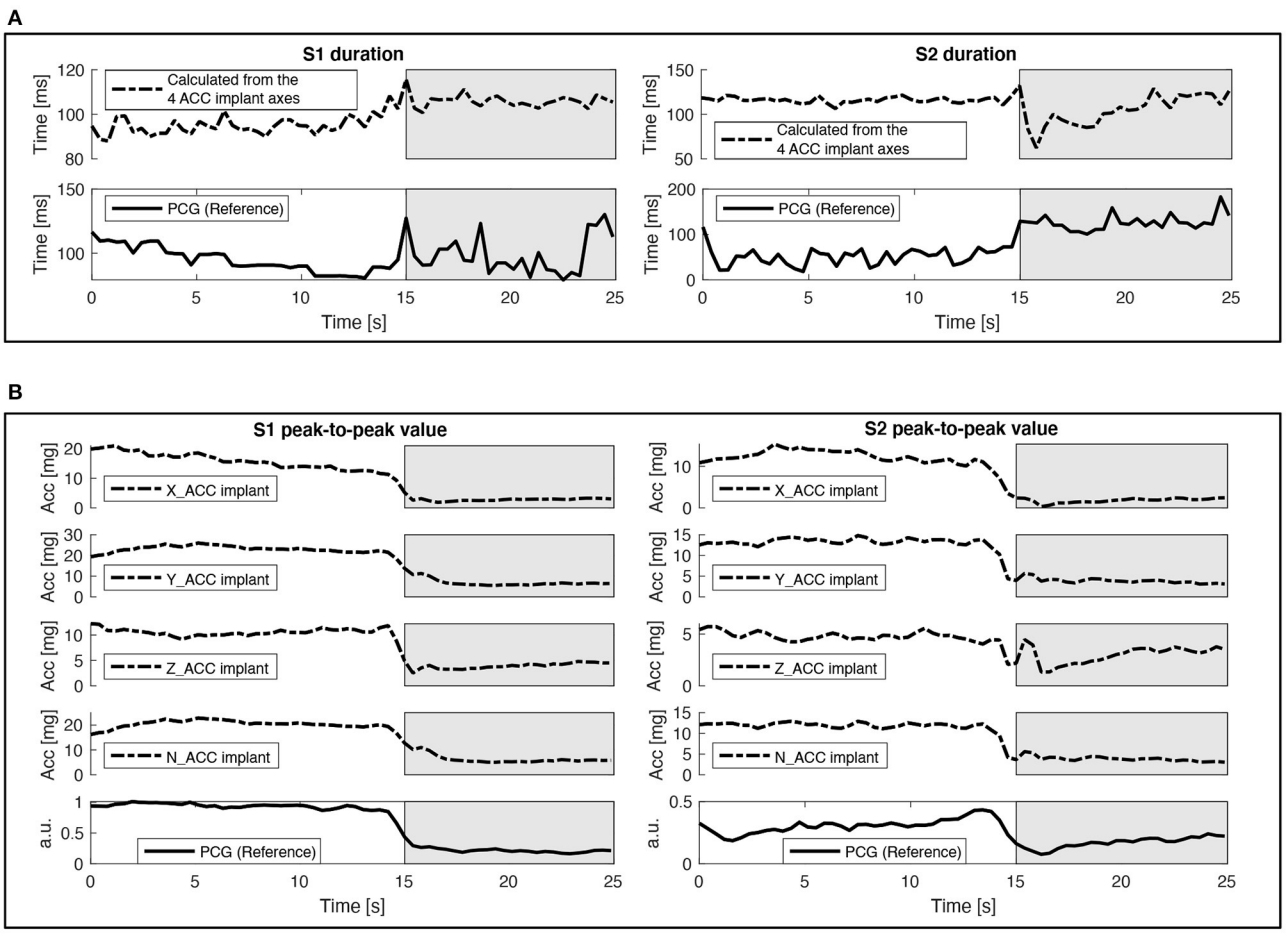
Example of implant (ACC) and gold standard (PCG) signals evolution over time. Dash-dotted lines correspond to the implant signals and solid lines correspond to the gold standard reference signals. The white background corresponds to the CPP stage and the gray background corresponds to the apnea stage. **(A)** Time-profiles of the duration of S1 and S2 measured on the recording 1. **(B)** Time-profiles of the peak-to-peak values of S1 and S2 measured on the recording 1.

Figure 6B shows coherent mean cardiac cycles of ACC and PCG (reference) signals that were band-pass filtered between 20 and 40 Hz. The reference PCG sensor observes cardiac vibrations in a different site, with a different angle and with a different transducer than the gastric site. This explains the differences in morphology between the PCG and ACC signals. However, from this first qualitative analysis we can hypothesize that the instant of occurrence of heart sounds, as well as the relative variation of their amplitudes or energies between the CPP and apnea phases could be correctly estimated from the gastric site. This evolution of the duration of S1 and S2 from the CPP to the apnea phases is presented in Figure 7A, while the evolution of the peak-to-peak amplitude is shown in Figure 7B. Similar dynamics can be observed between the ACC data acquired from the gastric site and the reference PCG. Nevertheless, it is possible to see how the similarity between time profiles is mainly perturbed during the transition moment between the CPP and apnea phases, more precisely, around the 16th second. This disturbance is caused by a sudden increase in the noise captured by the sensors during the transition moment between phases, and it is more evident over S2 in both duration and peak-to-peak values but mainly reflected over the Z-axis.

Table 2 shows quantitative results of the comparison between the ACC signals of the implant and the PCG reference. The correlation values between the time profiles of the duration of S1 and S2 are high and acceptable for both recordings, always presenting a higher correlation for S1 compared to S2. The algorithm to estimate the final detection instants of S1 and S2 by fusing the local detections from all the ACC axes of the implant is of utmost importance in these results because it allows estimating the global times of S1 and S2, even if it is not possible to detect one of the cardiac sounds in any axis. In the same way, the time profiles of the peak-to-peak values show high correlation values for all axes of the implant, and mainly for the Y and Z axes, showing how the measures performed by the implant are highly correlated with the PCG reference through the whole recording time. These results suggest that a suitable measuring of the variation of cardiac vibration signals can be performed using the ACC data from the implant.

**Table 2:** Validation of ACC recordings with a gold standard reference.

	**Heart** **sound**	**Correlation between the implant and the PCG reference**							
		**Recording 1**				**Recording 2**			
		**X**	**Y**	**Z**	**N**	**X**	**Y**	**Z**	**N**
Duration time-profile	S1	0.991					0.961		
	S2	0.874					0.950		
Peak-to-peak time-profile	S1	0.988	0.996	0.989	0.994	0.959	0.975	0.987	0.975
	S2	0.918	0.956	0.971	0.958	0.877	0.888	0.962	0.887

### 3.2. Estimation of Longitudinal Digital Markers From Gastric ECG and ACC Data

Table 3 shows the results of the recordings removed in each stage of the quality analysis, and the recordings kept to be analyzed in depth. These results show that it was possible to identify coherent cycles in most of the signals, having to remove just 1.8% of the recordings because they did not have enough coherent cycles at the ECG or ACC signals. This low rejection percentage reflects a good QRS detection process and correlation analysis, opening the possibility to perform basic heart rate variability analysis using this technology. Most of the removed recordings corresponded to stages 2 and 3, with rejection rates of 12.0 and 18.1%, respectively. These percentages mainly reflect the complexity to identify and suppress all the different types of noise sources present in the ACC data (such as pig growls and digestive sounds and movements), basically because stages 2 and 3 removed the recordings where the noise level is so high that it prevents detecting S1 and S2 correctly. After this quality assessment process, 68.1% of the total data were preserved. For these preserved recordings, the data quality allowed for a successful detection of S1 and S2 in the four axes, favoring the possibility of identifying useful and reliable hemodynamic markers from these signals by having more trustworthy information sources.

**Table 3:** Description of the data acquired for phase 2 and summary of the signal quality results.

**Pig** **ID**	**Implant** **version**	**Class**	**Time** **frame** **in days**	**Total** **number of** **recordings**	**Removed** **at stage** **1**		**Removed** **at stage** **2**		**Removed** **at stage** **3**		**Recordings** **finally** **preserved**	
					**#**	**%**	**#**	**%**	**#**	**%**	**#**	**%**
1	V1	Healthy	7	95	0	0.0%	18	18.9%	18	18.9%	59	62.1%
2	V1	Healthy	14	163	7	4.3%	22	13.5%	23	14.1%	111	68.1%
3	V1	Healthy	14	232	2	0.9%	16	6.9%	39	16.8%	175	75.4%
4	V1	Healthy	14	316	7	2.2%	40	12.7%	61	19.3%	208	65.8%
5	V2	Induc. HF	13	70	1	1.4%	10	14.3%	12	17.1%	47	67.1%
6	V2	Induc. HF	14	73	0	0.0%	4	5.5%	18	24.7%	51	69.9%
7	V2	Induc. HF	13	50	1	2.0%	10	20.0%	10	20.0%	29	58.0%
All	–	–	89	999	18	1.8%	120	12.0%	181	18.1%	680	68.1%

**Figure 8:**
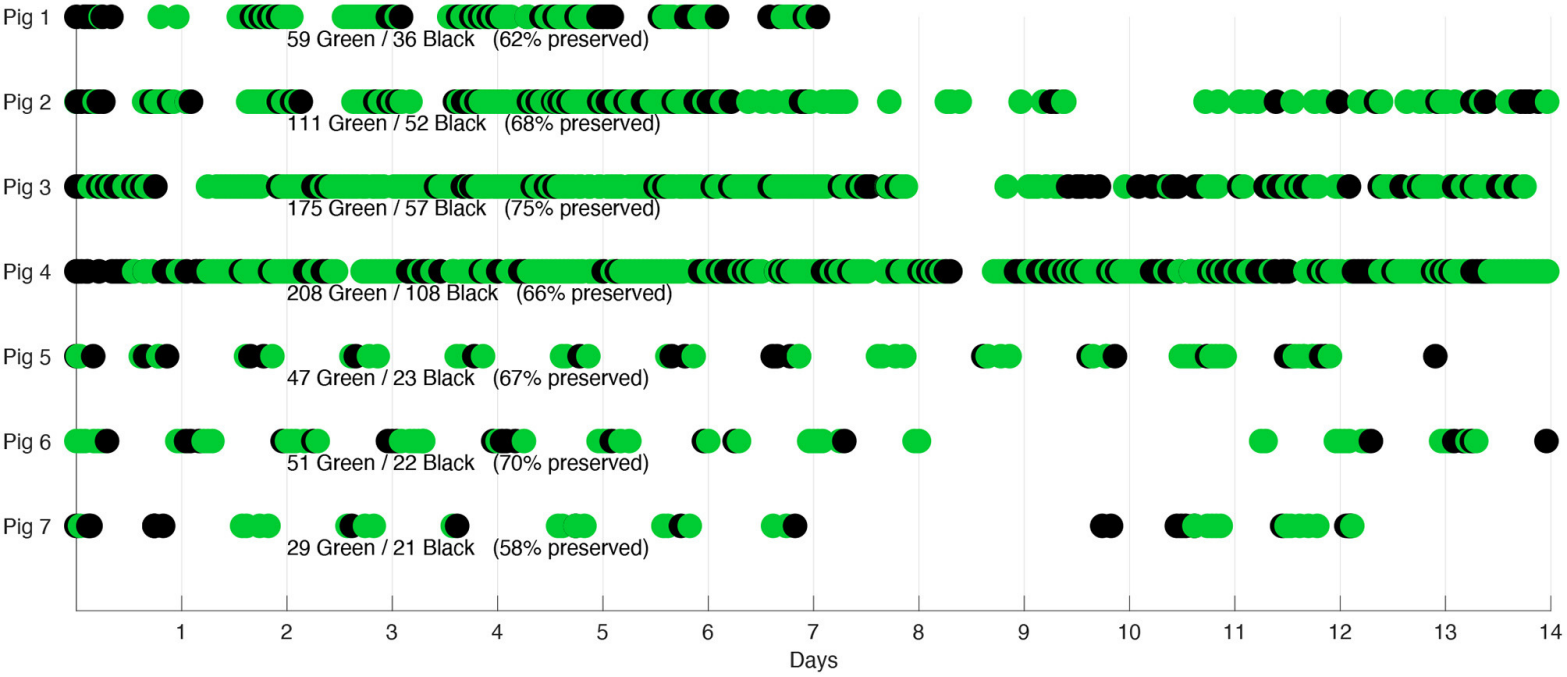
Signal acceptance distribution over time. Green dots represent the recordings finally preserved, black dots represent all discarded recordings.

Although these results show challenges to exploit a higher percentage of the acquired data, it is worth noting that continuous data acquisition along the day in the monitoring context of chronic diseases as HF is unnecessary. Figure 8 shows the distribution of rejected and preserved recordings over time, revealing that rejected signals are dispersed along all acquisition days for all the pigs, still allowing to preserve several useful recordings during each day. Furthermore, it is possible to see how the percentage of preserved recordings is higher than 60% for 6 of the 7 pigs regardless of whether they are healthy or pathological. Where the lowest percentage was obtained by the last pig (58%), but it is still comparable with the other pigs in terms of the distribution of preserved recordings over time, also considering that pig 7 had the fewest recordings available.

**Figure 9:**
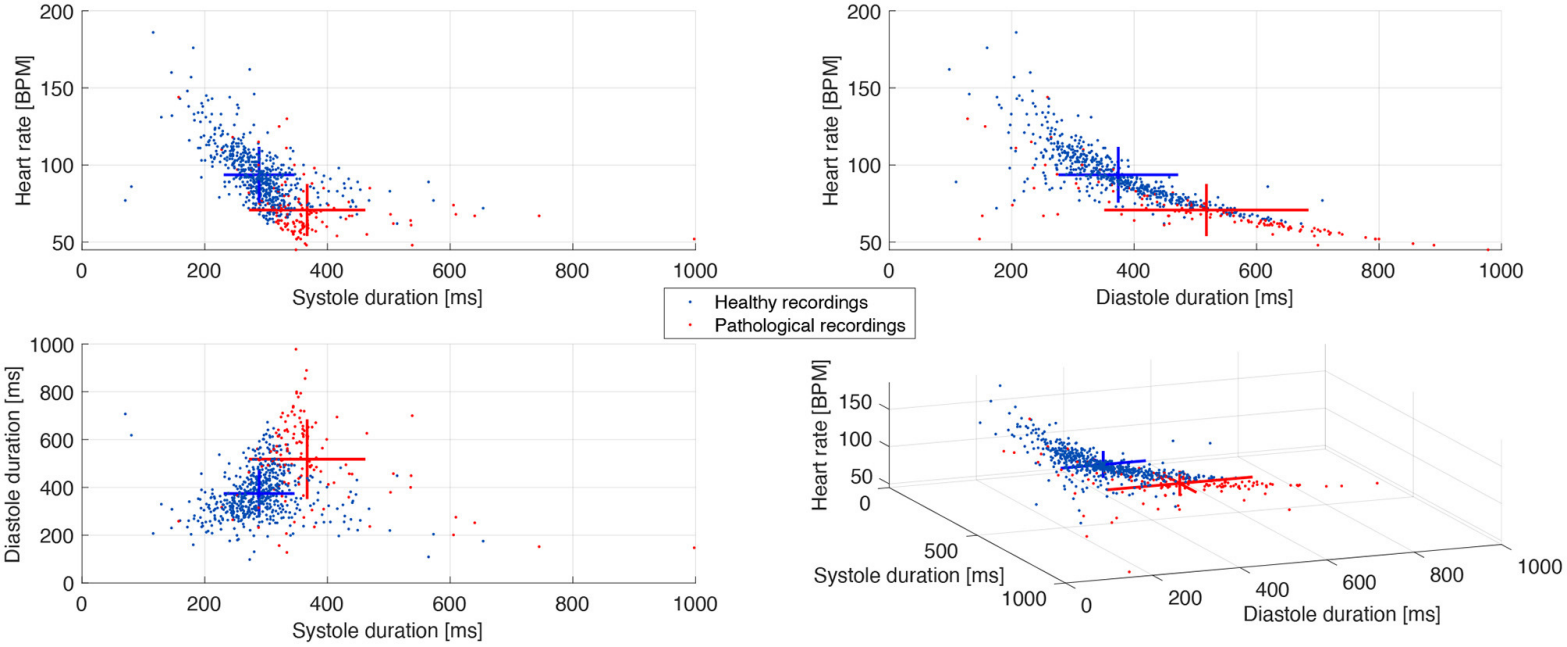
Scatter plot of the cardiac cycle duration between healthy and pathological pigs. The crosses represent the mean and standard deviation of each population along the three axes.

The estimation of the final detection instants of S1 and S2 allows to analyze the duration of the different cardiac cycle phases, such as the systole, diastole, and the S1 and S2 durations, where systole starts at *t*1 and ends at *t*3 and diastole corresponds to the total cycle duration minus the systole (Donal et al., 2011). Table 4 presents the mean and standard deviation of these variables for each pig. These results justify the procedure to detect S1 in the first half of the cardiac cycle because the mean duration of S1 is statistically lower than half of the mean total cycle duration for all pigs, assuring that S1 duration is not restricted by the search space. Additionally, it is possible to discern a pattern related to the HR between healthy and pathological pigs, where the 4 healthy pigs present a higher HR than the 3 pathological pigs. This pattern is better observed in Figure 9 through the scatter plot of the recordings relating the HR, systole, and diastole duration. A graphical representation of the mean and standard deviation of each one of these variables is also included. Although this pattern is evident in the data, it is not statistically supported to classify the pigs between healthy or pathological, considering all the characteristics that can influence the HRV as age, gender, weight, etc, and the limited number of pigs involved in the experiment.

**Table 4:** Statistics of cardiac cycle duration.

**Pig ID**	**Heart rate**	**Total cycle** **duration**	**Systole** **duration**	**Diastole** **duration**	**S1** **duration**	**S2** **duration**
	**[BPM]**	**[ms]**	**[ms]**	**[ms]**	**[ms]**	**[ms]**
1	92 ± 19	675 ± 124	291 ± 51	384 ± 102	168 ± 31	184 ± 38
2	89 ± 22	711 ± 155	297 ± 67	414 ± 124	152 ± 31	170 ± 41
3	92 ± 16	668 ± 99	297 ± 64	371 ± 93	142 ± 33	167 ± 42
4	98 ± 17	629 ± 95	276 ± 45	352 ± 76	153 ± 28	172 ± 36
5	77 ± 20	819 ± 174	366 ± 113	453 ± 149	189 ± 36	192 ± 49
6	63 ± 9	966 ± 132	358 ± 71	608 ± 137	199 ± 34	173 ± 34
7	74 ± 17	849 ± 169	383 ± 100	466 ± 175	199 ± 27	191 ± 43
All	89 ± 20	704 ± 155	303 ± 73	401 ± 127	159 ± 36	174 ±40

**Figure 10:**
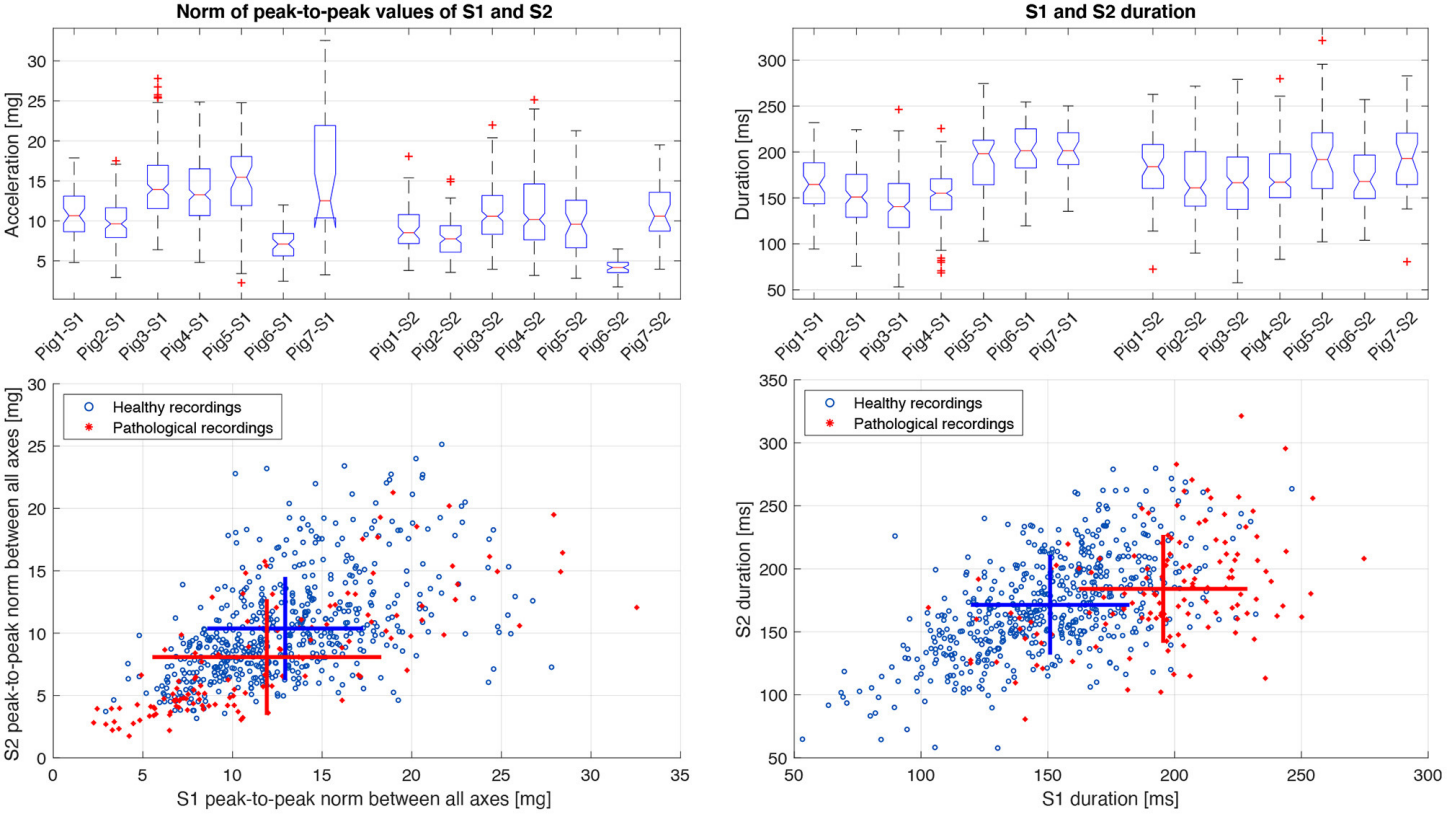
Statistical distribution and scatter plot of the norm of the peak-to-peak values between all ACC axes and the duration of S1 and S2 between healthy and pathological pigs. The crosses in the scatter plot represent the mean and standard deviation of each population.

The most relevant aspect to be analyzed about the estimation of hemodynamic markers, is the morphology of S1 and S2, i.e., the duration and amplitude of these cardiac components. Figure 10 shows through boxplots and scatter plots the statistical distribution of data according to the peak-to-peak values and duration of S1 and S2. Aiming to condense the information of the peak-to-peak values from the 4 ACC axes, it was decided to compute a magnitude measure by using the Euclidean norm of the peak-to-peak values from the 4 ACC axes. Additionally, this process ensures that even the recordings missing one or more of their ACC axes could be included in the analysis. Results in Figure 10 show that the median of peak-to-peak values of S1 are slightly higher than those of S2 for all pigs, which is consistent with the literature, both in PCG and SCG (Siejko et al., 2013; Ashouri et al., 2017; Sørensen et al., 2018). The scatter plot of peak-to-peak values suggests a reduction of the magnitude from the pathological recordings, and it is shown in S1 and S2. The difference between healthy and pathological pigs is more notable regarding the S1 and S2 duration, especially for S1, where the notches in the box plot do not overlap, concluding, with 95% confidence, that the true medians of S1 duration between healthy and pathological pigs do differ. These differences in peak-to-peak values and duration of heart sounds are expected between healthy and pathological pigs. Indeed, it has been previously reported that modifications of the inotropic state, which is particularly affected in HF, have a direct consequence on the amplitude and duration of S1 (Plicchi et al., 2002; Boehmer et al., 2017).

Table 5 shows the mean value and standard deviation of SNR measures from all cardiac cycles in the ECG and ACC signals. SNR measures obtained for the ECG signals show the highest values in the QRS-ECG configuration, as it was also presented with the SNR values of the gold standard signals in Table 1. Such phenomena are evident because the cut-off frequency values used for the QRS-ECG configuration were more restricted than the values used for the TP-ECG, suppressing in this way a bigger part of the noise in the signals and highlighting the R peak, which was the purpose of using both configurations in different stages of the processing pipeline. These values are also comparable with those found in the literature (Sahoo et al., 2016; Chatterjee et al., 2020).

**Table 5:** SNR of ECG and ACC signals.

**Pig** **ID**	**Signal-to-noise ratio (SNR) [dB]**									
	**ECG**		**S1**				**S2**			
	**TP**	**QRS**	**ACC_X**	**ACC_Y**	**ACC_Z**	**ACC_N**	**ACC_X**	**ACC_Y**	**ACC_Z**	**ACC_N**
1	14.7 ± 5.3	21.0 ± 4.0	13.2 ± 5.3	14.3 ± 5.5	14.3 ± 5.7	15.1 ± 5.4	9.7 ± 5.3	10.3 ± 5.4	10.1 ± 5.3	11.2 ± 5.4
2	17.3 ± 6.1	22.9 ± 5.1	15.3 ± 5.7	16.0 ± 6.1	15.7 ± 5.8	15.8 ± 6.0	11.0 ± 6.0	12.5 ± 6.1	11.6 ± 5.8	12.0 ± 6.0
3	15.8 ± 6.1	22.3 ± 4.3	14.9 ± 5.8	14.4 ± 5.5	15.9 ± 5.5	15.1 ± 5.5	10.6 ± 5.6	10.7 ± 5.8	11.4 ± 5.5	11.5 ± 5.9
4	16.2 ± 5.7	21.2 ± 3.8	14.5 ± 5.9	15.8 ± 5.8	15.5 ± 6.1	15.4 ± 5.8	9.8 ± 5.7	11.9 ± 6.4	11.9 ± 6.2	11.4 ± 6.0
5	15.8 ± 6.6	23.5 ± 5.7	15.4 ± 6.4	15.9 ± 6.4	16.7 ± 6.9	15.8 ± 5.8	10.8 ± 6.3	11.9 ± 6.8	12.2 ± 6.6	11.7 ± 5.8
6	22.2 ± 6.2	31.0 ± 6.3	17.7 ± 5.3	18.2 ± 5.5	17.9 ± 5.3	17.2 ± 4.7	13.7 ± 5.7	13.0 ± 6.2	11.8 ± 5.6	12.5 ± 5.1
7	21.6 ± 5.9	25.4 ± 5.3	16.7 ± 6.6	18.2 ± 6.7	17.9 ± 6.2	19.4 ± 6.3	13.1 ± 6.5	14.7 ± 7.3	13.7 ± 5.9	14.7 ± 6.2
All	17.6 ± 6.0	23.9 ± 4.9	15.4 ± 5.9	16.1 ± 5.9	16.3 ± 5.9	16.3 ± 5.6	11.3 ± 5.9	12.1 ± 6.3	11.8 ± 5.8	12.1 ± 5.8

SNR results from ACC signals represent a satisfactory signal quality when compared to the literature (Yu et al., 2013; Deborah et al., 2016; Cordero Álvarez, 2020). Although there are some differences in the equations used to compute the SNR in the ACC signals in the literature, the results can be comparable in terms of the ratio given in dB. The mean SNR of S1 is higher than the SNR of S2 because the absolute amplitude of S1 is usually higher than S2 and both values are measured against the same signal background noise.

## 4. Discussion

This paper presented, to our knowledge, the first characterization of electrophysiological and 3D accelerometer data acquired from the gastric fundus in pre-clinical experimentation. Results obtained from Phase 1 showed a satisfactory correlation levels between the markers obtained from the gastric implant and those obtained from surface, standard sites. In particular, the variation over time of markers as HR and cardiac sounds duration and amplitude were highly correlated with the reference, which is the main focus of the proposed device for long-term monitoring of chronic diseases such as HF (Cleland et al., 2006; Ponikowski et al., 2016; Yancy et al., 2017). Although these results were obtained from a limited number of observations, and may warrant further comparisons, we consider them sufficient to move forward to the feasibility study on Phase 2.

Results related to the estimation of longitudinal digital markers from the implant show that electrophysiological sensors contain a mix of different bioelectrical sources, mainly ECG, electromyographic, electrogastrographic, and impedance modifications at the electrode-tissue interface due to respiratory, gastric and general movement of the animal. In general, the SNR is high enough to perform robust QRS complex detection and basic HRV analysis from these electrophysiological signals. Concerning cardiac vibration signals, the main sources of noise are from the pig growls as well as digestive sounds and movements. These sources cause an abrupt reduction on the SNR and signal contrast of ACC data that may fall below 5 dB and 2 respectively. This fact is directly reflected in the high percentage of recordings rejected in the second stage of the quality evaluation process, which depend on a contrast level above 2. Respiratory movement and general motor activity of the pig are also observable, but can be more easily attenuated or canceled. A data quality assessment phase has thus been proposed to select a subset of the acquired data, containing exploitable information.

When SNR values higher than 6 dB or cardiac sounds contrast higher than 2 are observed, S1 and S2 components can be correctly segmented from the accelerometer signal and hemodynamic markers can be estimated from these data. The obtained values associated with the morphology of S1 and S2 agree with the literature in the field, regarding the duration (Schmidt et al., 2010; Giorgis et al., 2012) and the amplitude (Siejko et al., 2013; Dopierala et al., 2019) of both cardiac components. Furthermore, considering that previous studies have correlated the amplitude of S1 with the left ventricular *dP*/*dt* (Plicchi et al., 2002; Bordachar et al., 2008, 2011; Boehmer et al., 2017), it is worth noting that S1 showed a lower mean peak-to-peak value and a higher duration on HF pigs with respect to healthy pigs, indicating a possible negative inotropic function of HF pigs. Modifications between HF and normal pigs are also observed on S2. However, these results do not have any statistical significance at this stage and further preclinical evaluations have to be performed, using the exact same instrumentation on both groups. Nevertheless, the qualitative correspondence between the obtained results and those from the literature, highlights the feasibility to derive reliable and traceable markers related to the hemodynamic disturbances associated with HF from the gastric fundus.

In terms of information obtained from the implant, the change in orientation through time represents one interesting aspect, which was considered as a prospective source of information (results not shown in this paper). Since the device is properly fixed to the gastric fundus, so these changes in orientation are not related to movements of the device within the stomach (which would be a major source of error), but to movements of the coupling between the heart, diaphragm, and adjacent gastric structures. In addition to compensating for these movements, the accuracy of an implantable 3D accelerometer can be used to extract potentially valuable information. Further work is going in this direction. It is worth mentioning that some technical issues linked to the implant prototypes construction have to be approached in future versions. Some of the main identified problems are listed below:

- Sometimes the implant was restarted for reasons unrelated to the acquisition protocol and the schedule was no longer available.
- The application on the gateway was not robust enough. It was necessary to reboot the gateway to relaunch the application. If this action was performed too late, some data was lost.
- The cloud infrastructure was only in the first release for proof of concept and was not continuously stable.

## 5. Conclusion

This work shows initial, preclinical evidence on the feasibility of chronic cardiovascular monitoring from an implantable cardiac device located at the gastric fundus. The main challenge remains on the optimization of the signal-to-noise ratio, in particular for the handling of some sources of noise that are specific to the gastric acquisition site. Ongoing work is directed toward the proposal of adaptive methods that will activate data acquisition on the implant when specific noise-level criteria are met and on further preclinical evaluation.

## Data Availability Statement

The original contributions presented in the study are included in the article/supplementary material, further inquiries can be directed to the corresponding author/s.

## Ethics Statement

The animal study was reviewed and approved by Ethics committee recognized by the French Ministry of Research.

## Author Contributions

CD: data curation. AH, LS, FB, and PG: conceptualization. HA-L, AH, FB, and PG: formal analysis. PG and AH: funding acquisition. HA-L, CD, and AH: investigation and writing-original draft. HA-L and AH: methodology. HA-L: software. LS and AH: supervision. HA-L, CD, LS, FB, PG, and AH: validation and writing-review and editing. All authors contributed to the article and approved the submitted version.

## Funding

Results incorporated in this publication received funding from the ANR DIGS project (ANR-18-CE19-0008).

## Conflict of Interest

CD is employed by SentinHealth SA. The remaining authors declare that the research was conducted in the absence of any commercial or financial relationships that could be construed as a potential conflict of interest.

## Publisher's Note

All claims expressed in this article are solely those of the authors and do not necessarily represent those of their affiliated organizations, or those of the publisher, the editors and the reviewers. Any product that may be evaluated in this article, or claim that may be made by its manufacturer, is not guaranteed or endorsed by the publisher.

## References

[B1] Ashouri H. Hersek S. Inan O. T. (2017). Universal pre-ejection period estimation using seismocardiography: quantifying the effects of sensor placement and regression algorithms. IEEE Sensors J. 18, 1665–1674. 10.1109/JSEN.2017.2787628 29867294 PMC5983029

[B2] Boehmer J. P. Hariharan R. Devecchi F. G. Smith A. L. Molon G. Capucci A.. (2017). A multisensor algorithm predicts heart failure events in patients with implanted devices: results from the multisense study. JACC Heart Fail. 5, 216–225. 10.1016/j.jchf.2016.12.011 28254128

[B3] Bordachar P. Garrigue S. Ritter P. Ploux S. Labrousse L. Casset C.. (2011). Contributions of a hemodynamic sensor embedded in an atrial lead in a porcine model. J. Cardiovasc. Electrophysiol. 22, 579–583. 10.1111/j.1540-8167.2010.01930.x 20946232

[B4] Bordachar P. Labrousse L. Ploux S. Thambo J.-B. Lafitte S. Reant P.. (2008). Validation of a new noninvasive device for the monitoring of peak endocardial acceleration in pigs: implications for optimization of pacing site and configuration. J. Cardiovasc. Electrophysiol. 19, 725–729. 10.1111/j.1540-8167.2008.01105.x 18284498

[B5] Calvo M. Bonnet J.-L. Le Rolle V. Lemonnier M. Yasuda S. Oosterlinck W.. (2018). Evaluation of three-dimensional accelerometers for the study of left ventricular contractility, in 2018 Computing in Cardiology Conference (CinC), Vol. 45, (Maastricht: IEEE), 1–4.

[B6] Cao M. Gardner R. S. Hariharan R. Nair D. G. Schulze C. An Q.. (2020). Ambulatory monitoring of heart sounds via an implanted device is superior to auscultation for prediction of heart failure events. J. Cardiac Fail. 26, 151–159. 10.1016/j.cardfail.2019.10.006 31634574

[B7] Carrano F. M. Peev M. P. Saunders J. K. Melis M. Tognoni V. Di Lorenzo N. (2020). The role of minimally invasive and endoscopic technologies in morbid obesity treatment: review and critical appraisal of the current clinical practice. Obes. Surg. 30, 736–752. 10.1007/s11695-019-04302-8 31802407

[B8] Chatterjee S. Thakur R. S. Yadav R. N. Gupta L. Raghuvanshi D. K. (2020). Review of noise removal techniques in ECG signals. IET Signal Process. 14, 569–590. 10.1049/iet-spr.2020.0104

[B9] Cleland J. G. Daubert J.-C. Erdmann E. Freemantle N. Gras D. Kappenberger L.. (2006). Longer-term effects of cardiac resynchronization therapy on mortality in heart failure [the cardiac resynchronization-heart failure (care-hf) trial extension phase]. Eur. Heart J. 27, 1928–1932. 10.1093/eurheartj/ehl099 16782715

[B10] Cordero Álvarez R. (2020). Subcutaneous Monitoring of Cardiac Activity for Chronically Implanted Medical Devices (Ph.D. thesis). Université Paris-Saclay.

[B11] Deborah M. D. Prasad J. Aamina A. Devi A. R. (2016). Phonocardiogram signal processing using lms adaptive algorithm. Int. J. Multidiscipl. Approach Stud. 3, 66–73.

[B12] Delnoy P. P. Marcelli E. Oudeluttikhuis H. Nicastia D. Renesto F. Cercenelli L.. (2008). Validation of a peak endocardial acceleration-based algorithm to optimize cardiac resynchronization: early clinical results. Europace 10, 801–808. 10.1093/europace/eun125 18492682 PMC2435018

[B13] Desai A. S. Bhimaraj A. Bharmi R. Jermyn R. Bhatt K. Shavelle D.. (2017). Ambulatory hemodynamic monitoring reduces heart failure hospitalizations in real-world clinical practice. J. Am. Coll. Cardiol. 69, 2357–2365. 10.1016/j.jacc.2017.03.009 28330751

[B14] Donal E. Giorgis L. Cazeau S. Leclercq C. Senhadji L. Amblard A.. (2011). Endocardial acceleration (sonr) vs. ultrasound-derived time intervals in recipients of cardiac resynchronization therapy systems. Europace 13, 402–408. 10.1093/europace/euq411 21212110

[B15] Dopierala C. Gumérya P.-Y. Frikhaa M.-R. Thiébaulta J.-J. Cinquina P. Bouchera F. (2019). Digital implantable gastric stethoscope for the detection of early signs of acute cardiac decompensation in patients with chronic heart failure. Actes LAtelier Ia Sante.

[B16] Doyen M. Ge D. Beuchée A. Carrault G.I. Hernández A. (2019). Robust, real-time generic detector based on a multi-feature probabilistic method. PLoS ONE 14:e0223785. 10.1371/journal.pone.0223785 31661497 PMC6818956

[B17] Gallet C. Le Rolle V. Bonnet J.-L. Henry C. Hagège A. Mabo P.. (2016). Analysis of endocardial micro-accelerometry during valsalva maneuvers, in 2016 Computing in Cardiology Conference (CinC) (Vancouver: IEEE), 21–24.

[B18] Giorgis L. Frogerais P. Amblard A. Donal E. Mabo P. Senhadji L.. (2012). Optimal algorithm switching for the estimation of systole period from cardiac microacceleration signals (sonr). IEEE Trans. Biomed. Eng. 59, 3009–3015. 10.1109/TBME.2012.2212019 22893366

[B19] Gupta P. Moghimi M. J. Jeong Y. Gupta D. Inan O. T. Ayazi F. (2020). Precision wearable accelerometer contact microphones for longitudinal monitoring of mechano-acoustic cardiopulmonary signals. NPJ Digit. Med. 3, 1–8. 10.1038/s41746-020-0225-7 32128449 PMC7015926

[B20] Hasler W. (2009). Methods of gastric electrical stimulation and pacing: a review of their benefits and mechanisms of action in gastroparesis and obesity. Neurogastroenterol. Motil. 21, 229–243. 10.1111/j.1365-2982.2009.01277.x 19254353

[B21] Hernández A. I. Ziglio F. Amblard A. Senhadji L. Leclercq C. (2013). Analysis of endocardial acceleration during intraoperative optimization of cardiac resynchronization therapy, in 2013 35th Annual International Conference of the IEEE Engineering in Medicine and Biology Society (EMBC) (Osaka: IEEE), 7000–7003. 10.1109/EMBC.2013.6611169 PMC3900433 24111356

[B22] Inan O. T. Migeotte P.-F. Park K.-S. Etemadi M. Tavakolian K. Casanella R.. (2014). Ballistocardiography and seismocardiography: a review of recent advances. IEEE J. Biomed. Health Inform. 19, 1414–1427. 10.1109/JBHI.2014.2361732 25312966

[B23] Jain P. K. Tiwari A. K. (2014). Heart monitoring systemsa review. Comput. Biol. Med. 54, 1–13. 10.1016/j.compbiomed.2014.08.014 25194717

[B24] Plicchi G. Marcelli E. Parlapiano M. Bombardini T. (2002). Pea I and pea II based implantable haemodynamic monitor: pre clinical studies in sheep. Europace 4, 49–54. 10.1053/eupc.2001.0204 11846317

[B25] Ponikowski P. Voors A. A. Anker S. D. Bueno H. Cleland J. G. Coats A. J.. (2016). 2016 esc guidelines for the diagnosis and treatment of acute and chronic heart failure: The task force for the diagnosis and treatment of acute and chronic heart failure of the european society of cardiology (esc) developed with the special contribution of the heart failure association (hfa) of the esc. Eur. Heart J. 37, 2129–2200. 10.1093/eurheartj/ehw128 27206819

[B26] Sahoo S. Biswal P. Das T. Sabut S. (2016). De-noising of ECG signal and qrs detection using hilbert transform and adaptive thresholding. Proc. Technol. 25, 68–75. 10.1016/j.protcy.2016.08.08229801924

[B27] Šarlija M. Jurišić F. Popović S. (2017). A convolutional neural network based approach to qrs detection, in Proceedings of the 10th International Symposium on Image and Signal Processing and Analysis (Ljubljana: IEEE), 121–125.

[B28] Schmidt S. E. Holst-Hansen C. Graff C. Toft E. Struijk J. J. (2010). Segmentation of heart sound recordings by a duration-dependent hidden markov model. Physiol. Meas. 31:513. 10.1088/0967-3334/31/4/004 20208091

[B29] Shandhi M. M. H. Semiz B. Hersek S. Goller N. Ayazi F. Inan O. T. (2019). Performance analysis of gyroscope and accelerometer sensors for seismocardiography-based wearable pre-ejection period estimation. IEEE J. Biomed. Health Inform. 23, 2365–2374. 10.1109/JBHI.2019.2895775 30703050 PMC6874489

[B30] Siejko K. Z. Thakur P. H. Maile K. Patangay A. OLIVARI M.-T. (2013). Feasibility of heart sounds measurements from an accelerometer within an icd pulse generator. Pacing Clin. Electrophysiol. 36, 334–346. 10.1111/pace.12059 23252962

[B31] Sørensen K. Schmidt S. E. Jensen A. S. Søgaard P. Struijk J. J. (2018). Definition of fiducial points in the normal seismocardiogram. Sci. Rep. 8, 1–11. 10.1038/s41598-018-33675-6 30337579 PMC6193995

[B32] Thakur P. H. An Q. Swanson L. Zhang Y. Gardner R. S. (2017). Haemodynamic monitoring of cardiac status using heart sounds from an implanted cardiac device. ESC Heart Fail. 4, 605–613. 10.1002/ehf2.12171 29154421 PMC5695191

[B33] Yancy C. W. Jessup M. Bozkurt B. Butler J. Casey D. E. Colvin M. M.. (2017). 2017 acc/aha/hfsa focused update of the 2013 accf/aha guideline for the management of heart failure: a report of the American College of Cardiology/American Heart Association task force on clinical practice guidelines and the heart failure society of America. J. Am. Coll. Cardiol. 70, 776–803. 10.1161/CIR.0000000000000509 28461007

[B34] Yu Z. Bui F. M. Babyn P. Dinh A. (2013). Evaluation of compressed sensing in seismocardiogram (SCG) systems, in 2013 26th IEEE Canadian Conference on Electrical and Computer Engineering (CCECE) (Regina: IEEE), 1–4.

